# Probability of consolidation constrains novel serotype emergence in dengue fever virus

**DOI:** 10.1371/journal.pone.0248765

**Published:** 2021-04-05

**Authors:** Gilberto Sánchez-González, Zachery R. Belak, Luis Lozano, Renaud Condé

**Affiliations:** 1 Centro de Investigación Sobre Enfermedades Infecciosas, Instituto Nacional de Salud Pública, Cuernavaca, Morelos, México; 2 ZYUS Life Sciences Inc., Saskatoon, Saskatchewan, Canada; 3 Centro de Ciencias Genómicas, Universidad Nacional Autónoma de México, Cuernavaca, Morelos, Mexico; University of Padua, ITALY

## Abstract

Since their first sequencing 40 years ago, Dengue virus (DENV) genotypes have shown extreme coherence regarding the serotype class they encode. Considering that DENV is a ribonucleic acid (RNA) virus with a high mutation rate, this behavior is intriguing. Here, we explore the effect of various parameters on likelihood of new serotype emergence. In order to determine the time scales of such an event, we used a Timed Markov Transmission Model to explore the influences of sylvatic versus peri-urban transmission, viral mutation rate, and vertical transmission on the probabilities of novel serotype emergence. We found that around 1 000 years are required for a new serotype to emerge, consistent with phylogenetic analysis of extant dengue serotypes. Furthermore, we show that likelihood of establishing chains of mosquito-human-mosquito infection, known as consolidation, is the primary factor which constrains novel serotype emergence. Our work illustrates the restrictions on and provides a mechanistic explanation for the low probability of novel dengue virus serotype emergence and the low number of observed DENV serotypes.

## Introduction

Rates of dengue virus (DENV) infection have shown a marked increase during the last two decades, imposing a sizable burden on the health systems and economies of affected Latin-American countries [1]. Current vaccine prospects provide limited infection protection efficacy (from 42.3% to 77.7% protection, depending on the serotype), but are drastically decreasing the appearance of hemorrhagic symptoms in the infected population [2]. Part of the difficulty for dengue vaccine design arises from the existence of 4 distinct dengue virus serotypes, each with multiple genomic sequences. Despite the existence of thousands of genotypes for each serotype, the viruses maintain high homology between their structural protein sequences [3]. This phenomenon contrasts with human papillomavirus (a DNA virus), where observed mutations have given rise to over 170 serotypes [4]. The occurrence of DENV viruses not belonging to the 4 previously described serotypes has been reported in Malaysia, but this newcomer virus did not further propagate in the population [5]. The envelope (E) protein mediates virus binding to and fusion with the host cell membrane [6]. Immune response against this protein generates neutralizing, anti-fusion, as well as replication-enhancing antibodies [3]. The soluble portion of the Envelope (E) protein is composed of three distinct domains, designated I–III. E protein is present as a dimer on the viral surface and domain I organizes this structure. Domain II bears the fusion peptide required for viral-cellular membrane fusion under low pH conditions, and its central loop is the sequence responsible for eliciting the greatest part of the immune response [7]. Domain III has an immunoglobulin-like structure and is primarily responsible for binding to cellular receptors via a region adjacent to domain I known as the E III lateral ridge [8–10]. The E protein domain III lateral ridge is the core of the serotype-specific sequences that are conserved within each DENV serotype, and are therefore one of the main serotype defining epitopes of the virus [10]. DENV phylogeny based on RNA sequences usually presents the following branching order: the first divergence corresponds to DENV-4, then to DENV-2, and the final branching occurs between DENV-1 and DENV-3. Alternatively, a NS-5 based phylogenetic tree groups DENV-2 with DENV-3 (though with weak bootstrap support) and shows DENV-1 clustering with DENV-4 [11].

Sylvatic transmission of DENV serotypes 2 and 4 has been regarded as the setting for emergence of the DENV-1 and DENV-3 serotypes, the supporting observation being the identification of sylvatic transmission cycles of DENV-2 and DENV-4 in monkeys in Asia and West Africa [12–14]. The RNA sequences of DENV-2 and DENV-4 sylvatic strains fall basal to the human dengue viruses within their respective serotype, suggesting DENV strains isolated from humans originated from the sylvatic environment [15]. Although no sylvatic strains have yet been identified in DENV-3, the presence of DENV-3 antibodies in Malaysian monkeys suggests that a sylvatic cycle also exists for this serotype [16]. No DENV-1 sylvatic strains transmission has been observed in Africa, and this strain remains closely associated with *Aedes aegypti* [17], though *Ae*. *taylori* laboratory infections with DENV-1 were marginally effective. Another set of experiment showed that neither *A*. *luteocephalus* or *A*. *taylori* sylvatic mosquitoes could be infected by DENV-1 [15]. Hence, there is some evidence that dengue was originally a virus infecting forest mammals and that cross-species transmission to humans has occurred independently in all four serotypes [11]. Conversely, it has been shown that urban/human DENVs infect forest animals (monkeys) and are sustained there before eventually returning to the urban ambit and infecting a human host [18]. These observations have given rise to the current widespread belief that simple viral mutation during sylvatic transmission is the major phenomenon governing the probability of novel DENV serotype emergence.

In order to perpetuate infection, dengue viruses are compelled to pass from one host species to another. Other than minor transovarial transmission among mosquitos, dengue virus transmission follows a human > mosquito > human pattern, with human to human transmission absent from the viral lifecycle. Their ability to replicate in the cells of different animal genera and different cell types within each animal is key to this cycle of alternate host infection. In humans, the virus is able to infect liver endothelial cells, lung vascular endothelial cells, kidney tubule multinucleated cells, spleen reactive lymphoid cells, macrophages, monocytes and lymphocytes [19]. In the insect (mostly mosquitoes of the *Aedes* genus), the viral particle mainly infects intestinal, circulatory and salivary gland cells [20]. Infection in both mammalian and insect hosts requires interactions of the viral E protein with specific cellular receptors, imposing structural constraints and ultimately mutational restrictions in certain portions of the E protein sequence [21]. While the need for the E-protein structure to be preserved is a key constraint on the ability of novel serotypes to emerge, this restriction alone does not explain the low rate of novel DENV serotype emergence. The four known DENV serotypes are only between 65% and 82% identical in their E-protein amino acid sequences, implying that serotype-modifying E-protein mutations should be easily functionally accommodated [11].

The virus must also cope with the immune system of its hosts. This constitutes another point of restriction that applies positive selection to virus capable of evading the host immune response. In humans, the evolutionary pressure on the virus is mainly attributable to the host’s adaptive immune response. Consequently, differences in E protein observed between DENV serotypes primarily modify the protein sequence of the receptor-interacting domain III of the viral E-protein. Interaction of domain III with the cellular receptor is a key step in infection, therefore neutralizing antibodies that block receptor-viral interaction are important in preventing cellular entry [7]. Analyses have shown that nucleotide variation represented only 5% of the viral genome between serotypes, but these changes were highly localized to regions of the E-protein responsible for receptor interaction and cell membrane fusion, indicating that the mutations needed for immunological evasion correspond to regions of the viral protein required for cell entry [22]. The significance of immunological evasion is substantial, given that when mutations allow a virus to escape previous immunity, the mutant could provoke severe dengue symptoms [23, 24].

Taken together, these observations would suggest a strong positive selection pressure on viral strains with altered serotype as they would have greater chance of immune evasion, yet paradoxically, the number and rate of novel serotype emergence among DENV is low.

In the insect, the main constraints affecting viability of viral mutants are associated with its ability to escape the mosquito RNA interference system [25, 26]. Additional restrictions on mutant viability are imposed by susceptibility to other immune defense mechanisms, such as protease and thioester protein mediated processes, but the viral genetic elements affected by the resulting evolutionary pressures remain unclear [27]. Importantly, while viral replication is underway in the insect vector, evolutionary pressure to maintain serotype category epitope structure is reduced, potentially increasing the likelihood of sufficient alterations to these sequences to enable immunological evasion upon re-infection of mammalian hosts. Regarding the timeframe during which the virus acquires mutations enabling escape from the vertebrate adaptative immune system, we must consider that the dengue virus can persist in the mosquito host through vertical transmission [28]. When transovarially infected, the mosquitoes increase their larval stage duration and diminish their fecundity and fertility [29], hence restricting the expansion of mosquito strains able to transmit the virus vertically. It is noteworthy that transovarial dengue transmission does increase the time during which the virus E protein can mutate without confronting the adaptative immune system of its vertebrate host. Again, these observations should act to increase the number and probability of emergence of DENV serotypes, in contrast the few serotypes actually observed.

DENV serotype specificity is determined by epitopes present in the protein motifs of the domain III lateral ridge of the E protein [10]. Therefore, the emergence of a new serotype will depend on the alteration of these epitopes. For this event to occur, the mutation must generate a viable virus with a significantly altered epitope sequence. The number of mutations required to render the virus particle inactive has been studied informatically by Burke et al. and was found to be inversely dependent on the proposed genome length [30]. The number of errors generated during DENV virus genome replication depends on the fidelity of its viral RNA-dependent RNA polymerase, NS5. The common error rate of RNA based RNA polymerases is 1 base every 10 000 bases replicated. Therefore, a large proportion of DENV particles are defective in part of their genome, both when recollected from human patients or cultured insect cells [31]. This fact explains the differences observed between DENV quantitation by polymerase chain reaction (PCR), by focus formation unit, and by electron microscopy. Quantities of functional DENV, as measured by focus forming unit, are overestimated 10 times when measured by electron microscopy and 10 000 times when assessed by PCR [32]. Alternate virus passages from human Huh-7 cells to monkey Vero cells decreased virus infectivity, while passage in mosquito C6/36 cells resulted in variable changes in fitness (increases and decreases). This demonstrates that infection of mosquito cells imposes fewer constraints on the DENV sequence than vertebrate passage, thereby allowing the virus to gain diversity when passing through the insect host [33]. When analyzing the field mutation rate of DENV, Khan and al. observed that the yearly number of non-synonymous mutations ranged from 12 to 47 (0.35% to 1.39% of the genome). This mutation rate allows for substantial protein sequence variations and epitope alteration over a short time period [34]. These factors should further increase the rate of novel serotype emergence, however, as noted, actual appearance of novel serotypes of DENV is very rare.

Given the diverse phenomenon which logic dictates should act to increase the probability of novel serotype emergence, we wished to study further the possible reasons for observed rarity of novel DENV serotype appearance. We hypothesized that the emergence of a new serotype must be constrained by successive probabilistic events to explain the resilience of the serotype classes to drastic mutation. Here, we use a simple Timed Markov Model for DENV transmission to analyze the ratio of probability of introducing a peri-urban DENV strain to the sylvatic environment versus the reverse, always considering humans as the host vector. We estimate the possibility of emergence and establishment of a new DENV serotype, considering a closed urban transmission dynamic. We then examine the influence of viral mutation rate, timescale, and the effect of vertical transmission on probability of novel serotype emergence. The results of our analysis unexpectedly suggest that the peri-urban environment is a major site of viral evolution and that vertical transmission has little influence on the probability of novel serotype emergence. Having emerged, the new virus must pass several probabilistically challenging phases before becoming established both in the human and mosquito populations, a process known as consolidation. Our key findings are that novel serotype transmission events are common but novel serotype consolidation is the major probabilistic hurdle to emergence of new DENV serotypes. Together, these findings explain the low observed number of DENV serotypes, and the low number of serotypes present in flaviviruses generally. Our results aid in understanding the transmission dynamics of dengue Fever Virus with applications in viral control and mitigation, as well as facilitating a deeper understanding of the origins of novel viruses of concern to human and animal health.

## Material and methods

### General mathematical model and algorithm for mutation rate

A Timed Markov Transmission Model was developed to simulate the transmission dynamics between humans and mosquitoes. One of the defining attributes of Ordinary Markov Models is that the transition rates are constant and therefore the transitions are continuous. However, in our actual situation, a state transition that depends explicitly on time is required. These are provided by the Timed Markov Models. In these models, we want to construct a continuous time process on some countable state-space *S* that satisfies the Markov property $P(X_{n}=x_{n}X_{n-1}=x_{n-1})$, were *X*∈*S*, and $X={\left\{X_{n}:\Omega \to S\right\}}_{n\in N}$, conveying the stochastic process for the transition within the state-space *S*. The Timed Markov Models are defined as [35]:

$$P(X_{n}(t+h)=x_{n}|X_{n}(t)=x_{n})=T(x_{n},t+h)$$

$$P(X_{n}(t+h)=x_{n}|X_{n-1}(t)=x_{n-1})=T(x_{n},x_{n-1},t+h)$$

Where *T*(*x*_*n*_,*t*+*h*), and *T*(*x*_*n*_,*x*_*n*−1_,*t*+*h*) are the stochastic transition rates, evaluated at time *t + h*. Since, in our model, the system does not express the loss of memory property (because the epidemiological state of the system can be known in any moment and thus can be compared to another similar system with different elapsed time), the stochastic transition rates *T* cannot be (in general) an exponential distribution.

For each mosquito, we implemented an algorithm that introduces the mutation probability to the model. The stochastic process of new serotype occurrence is determined by multiplying the mutation rate per base by the RNA sequence length of the E-protein (1.5 Kb approximately) and monitoring the accumulated percentage of changes relative to the original sequence. The accumulated number of mutations is stochastic (i.e. time-dependent) while the total number of amino acids changed per mutation event is probabilistic. In our model, we established that amino acid sequence identity between 40%-80% relative to the parental strain would be designated a new serotype. This is a highly conservative estimate to ensure that the resulting E-protein would indeed constitute a novel serotype, as even the most divergent DENV strains possess ~65% amino acid identity between their E-protein sequences [36]. We employed the E-protein mutation rate determined by Hapuarachchi et al. (1.33x10^-3^ per site/per year) divided by 365 to establish the daily rate of antigen transformation [37], thus the probability of a new serotype occurrence is calculated as probability = time*daily mutation rate*E-protein length*sequence identity cutoff*proportion of infective virus. The stochastic determination of new serotype emergence is achieved through a Metropolis–Hastings algorithm [38].

Finally, some important points have to be considered for the calculation algorithm: in Table 1, numerical parameters are used to define probabilistic Beta Distributions, whose mean magnitudes correspond to the values of the parameter (with 10% standard deviations). Secondly; the parameters represented as functions (for example the EIP function) will be multiplied by 1+ N(0,0.1), were N represents a probabilistic Normal Distribution with a mean value of zero and standard deviation of 10%. This factor is a dispersion function that introduces variability to the parameter. The calculations of the model were performed using the TreeAge Pro 2008 program, which is a Python-based platform used to perform random walks.

**Table 1:** Values and interpretation of the parameters of the model.

Parameter interpretation	Value	Reference
Human population sample [H]	100 000 per sample	Proposed
Mosquitoes population sample [M]	100 000 per sample	Proposed
Recalculations (loops)	500	Proposed
Initial proportion of urban infected mosquitoes	20%	[40]
Initial proportion of infected humans	0	Supposed
Infectious meal rate from humans to mosquitoes	0.3	[41, 42]
Mortality rate of uninfected mosquito [k]	(−90.66+9.54 T−0.18 T^2^)^−1^	[43]
Mortality rate of infected mosquito	1.56 k	[44]
Infectious bites rate from mosquitoes to humans	0.2	[42, 43, 45]
Immunity acquisition rate [R(t)]	0.14	[46]
Immunity loss rate (daily loss)	0.001%	[47]
Extrinsic Incubation Period [EIP]	$600\sqrt{\frac{0.3}{2\pi }}\;Exp\left(-0.3\frac{{\left(T-5.9\right)}^{2}}{T}\right)$	[48]
Cross protection duration	1–2 weeks	[49]
Mutation rate per amino acid per year	0.35% to 1.39%	[33]
E-protein length	480 amino acids	[50]
Proportion of infective virus	10^−4^	[32]
Percentage of sequence identity to define new serotype	40%-80%	[11, 36, 51, 52]
Percentage of persons which enters to the forest to carry out their daily activities	30%–90%	Supposed
Proportion of symptomatic dengue infection in humans	4.2%	[53]
Proportion of sylvatic mosquitoes infected with DENV	1.13%	[54]
**Explicit Calculations**		
Dynamic of human-infected population [I(t)]	$I(t)=\frac{NHI(t-1)+I(t-1)-NRH(t-1)+NSTE(t-1)}{H}$	
Dynamic of human-infected population [R(t)]	$R(t)=\frac{R(t-1)+NRH(t-1)-HLI(t-1)}{H}$	
Dynamic of susceptible-human population [S(t)]	*S*(*t*) = 1−[I(t−1)+R(t−1)]	
Dynamic mosquito-infected population [J(t)]	$J(t)=\frac{NMI(t-1)+J(t-1)-DIM(t-1)+NSCE(t-1)}{M}$	
Temperature	$25+5\;{Sin(\frac{t}{4*30}\pi )}^{2}$	

T = Temperature, t = Time, NSTE = New serotype transmission event, NHI = New human infection, NRH = New recovered human, HLI = Human immunity loss, NMI = New mosquito infection, NSCE = New sequence consolidation events, DIM = Death of infected mosquito.

### The peri-urban-sylvatic exchange simulation

**Fig 1:**
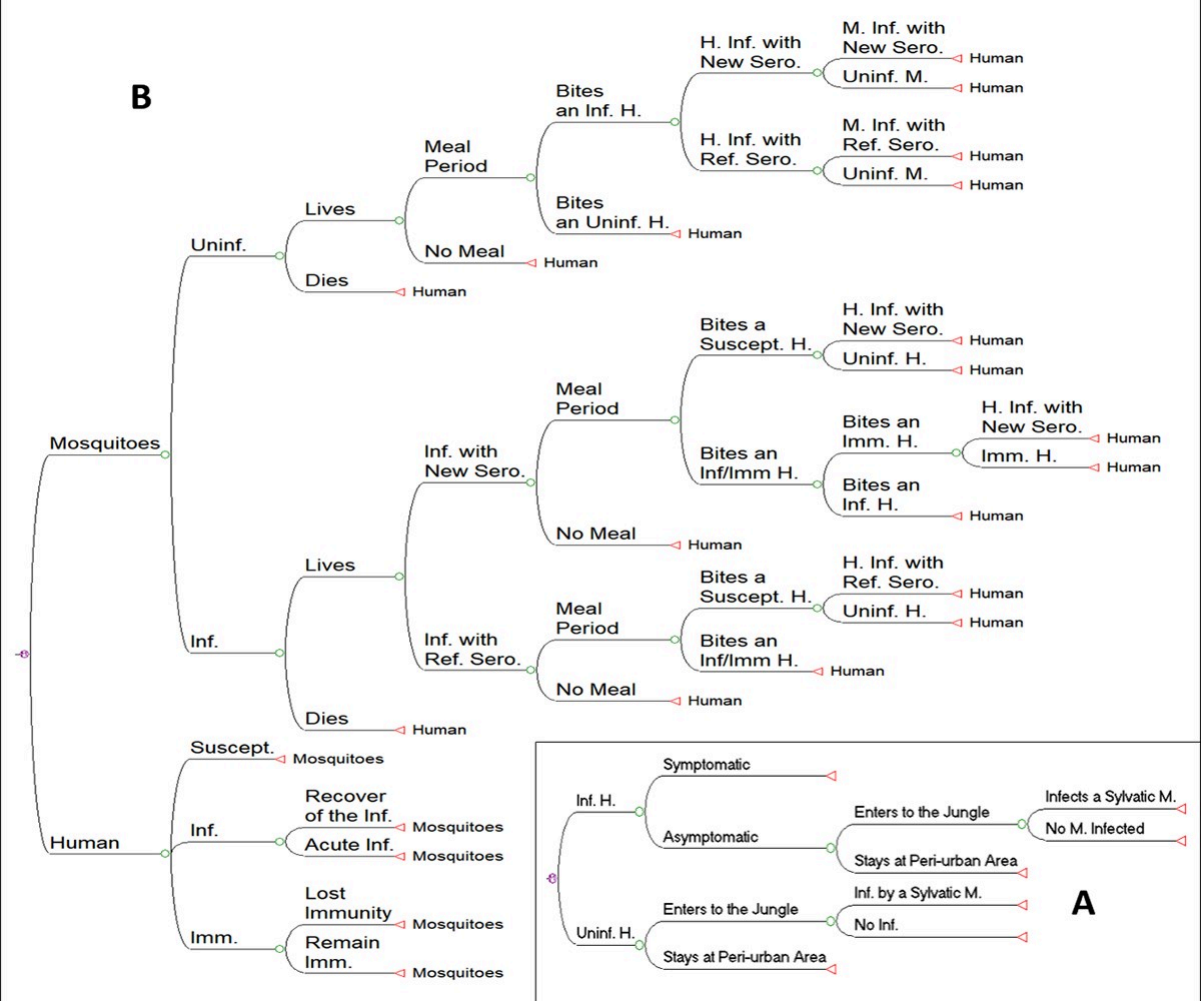
Graphical representation of the models A) Schematic of the Markov Model of the decision tree to estimate the peri-urban ↔ sylvatic probability transmission ratio. B) Schematic of the Markov Model. The nodes represent the events of immunological break and the events of new serotype establishment which are indicated with arrows. Abbreviations: Inf = Infected, Uninf = Uninfected, H = Human, M = Mosquito, Ref = Reference, Imm = Immune, Ser = Serotype, and Suscept = Susceptible.

This simulation assesses the peri-urban ↔ sylvatic transmission probability ratio, a representation of the probability of a sylvatic DENV strain being introduced to a peri-urban area. The simulation is a function of the human population that inhabits the peri-urban locations. Some proportion of this population presents a DENV infection of urban or peri-urban origin. Some individuals from this infected population will be symptomatic, hence considered as inactive during the illness period. Conversely, the asymptomatic infected population will behave normally; some of them going into the forest to perform their usual activities. During their time in the forest, these persons can be bitten by a sylvatic mosquito and the mosquito can acquire a DENV of urban or peri-urban origin. In parallel, a proportion of human uninfected population will enter the forest and face the risk of being bitten by a mosquito infected with a sylvatic strain of DENV. These individuals may get infected by this DENV strain and introduce it into their urban communities. The model simulated the transmission of DENV amongst 100 000 humans and monitors the evolution of epidemiological variables. This procedure was repeated 500 times, with different distribution samplings. After each simulation, the probability of peri-urban ↔ sylvatic transmission was computed. This simulation supposes similar abundances of mosquitos per person in the peri-urban area and in the forest (see the model schematic at Fig 1A).

The number of infections in humans and mosquitoes were counted, and the probabilities readjusted at each time step. We proceeded identically with the number of infection recoveries, the TNSEs and the NSCEs. For the first model approach, we modeled pluviometry of the rain season as a sine function with a 4-month period, where the temperature changes concomitantly from 25°C to 30°C. These temperatures cross the optimal transmission temperature which ranges between 27–28°C, as showed previously [39]. The parameters used to calculate the model are shown on Table 1.

### Simulation of urban environment transmission without sylvatic fringe

To estimate the time horizon for a new DENV serotype to emerge from exclusively urban transmission dynamics, we modeled the evolution using only the reported natural mutation rate of the virus. Our simulation postulates that the total population of humans and mosquitoes are at equilibrium, according to predator-prey rules [55]. The model evaluates two main branches, one for humans and the other for mosquitoes. The Human and mosquito infection rates are determined from the perspective of the mosquito branch. The immune conditions of the human branch are split into three states, which are: susceptible, acutely infected, and immune. In this model, the acutely infected state corresponds to acute infection presenting high levels of viremia, independent of the case symptomatology. The mosquito branches consist of uninfected and infected states, respectively. When computing the virus transmission rate, the model first considers the probability of death of infected mosquitoes. It then evaluates the probability of the surviving mosquitoes producing virus with a new serotype or of the infecting virus to remain within the consensus sequence. Once this condition is determined, the virus may be transmitted if the mosquitos enter a meal search period. If a mosquito carrying a consensus sequence bites a susceptible human, the human faces the risk of getting infected; though acutely infected or immune persons cannot be infected. When a mosquito carrying a new serotype bites a human, this human faces the risk of getting infected, even if it had previous immunity to DENV. This event is counted as a “transmission of a new serotype event” (TNSE) by the model. Given that this new virus will not be recognized by prior serotype specific anti-DENV antibodies, the mosquitoes carrying the new serotype DENV will have the ability to infect immune persons. Since we take into account cross-protection mechanism effects, this new re-infection will only be possible if the infected human has no cross-serotype protection. To complete the transmission cycle, a mosquito must bite a person infected with the emerging serotype and get infected. This event is hereafter counted as a “new serotype consolidation event” (NSCE). The probability for the mosquito to bite an infected person is proportional to the dengue incidence in the human population, therefore determined the number of infected humans at each time step of the model.

In the model, for each mosquito that dies, a new mosquito emerges; either naïve or infected, depending on the scenario (with or without transovarial transmission). In the human branch of the decision diagram, the susceptible persons remain susceptible until infected from the mosquito branch. When infected, they enter the acute period, where they remain for a period of time, until they probabilistically enter the immune state. Long-term immunological protection is lost after 2 years in 75% of the patients [47] (allowing the human to return to the susceptible state) or when a new serotype infects the subject when the poly-immunity is lost [49] (one or two weeks).

The model evaluates the infected states of the humans and the mosquitoes concurrently. To produce the alternate changes between infected populations, we force the model to enter the human branch once the mosquito branch has been evaluated. This allows determination of the human disease response status at the same time as the mosquito one. The time step size of the model is one day. The model evaluates and averages the results of simulating the interaction of 100 000 humans and 100 000 mosquitoes during a single rainy season (4 months modeled as a sine-square function). To provide the final probability of novel serotype emergence this procedure is repeated 500 times, using parameter values obtained from a random sampling of probabilistic distributions (see the model scheme in Fig 1B). This calculation approach (known as a second-order Monte Carlo Simulation) is fully compatible with a multivariate sensitivity analysis because it does also explore the variability of the parameters in each sampling of the distribution [56]. This methodology is equivalent to the bootstrapping method which infers the population mean based on sample data. It is a resampling method whereby the precision of sample statistics is estimated by independent sampling with replacement from an existing sample dataset with same sample size [57].

### Scenarios for the simulation of urban environment without sylvatic fringe

To investigate the effect of time and vertical (i.e. transovarial) transmission on new serotype consolidation in the population, multiple scenarios were evaluated. All the scenarios were calculated from 500 samples of the population, with 100 000 persons and 100 000 mosquitoes in each sample. In order to simulate an increase in observation time scale, the mutation probability was increased. Four scenarios were considered:

1. Basic scenario. The mutation rate used is the natural rate, and no vertical transmission of the virus is considered. The results are interpreted as the outcome of the transmission and mutation dynamics over one rainy season (one year).
2. The mutation rate is increased 100 times, with no vertical transmission. The results are interpreted as the outcome of the transmission and mutation dynamics over 100 rainy seasons (100 years).
3. The mutation rate is increased 1 000 times, with no vertical transmission. The results are interpreted as the outcome of the transmission and mutation dynamics over 1 000 rainy seasons (1 000 years).
4. The mutation rate is fixed as 1 000 times greater than the natural rate, and the proportion of vertical transmission are established at 10%, 30% and 50%. These calculations allow us to explore the effect of vertical transmission on new serotype consolidation.

## Results

### Dengue virus transmission is mainly peri-urban to sylvatic

**Fig 2:**
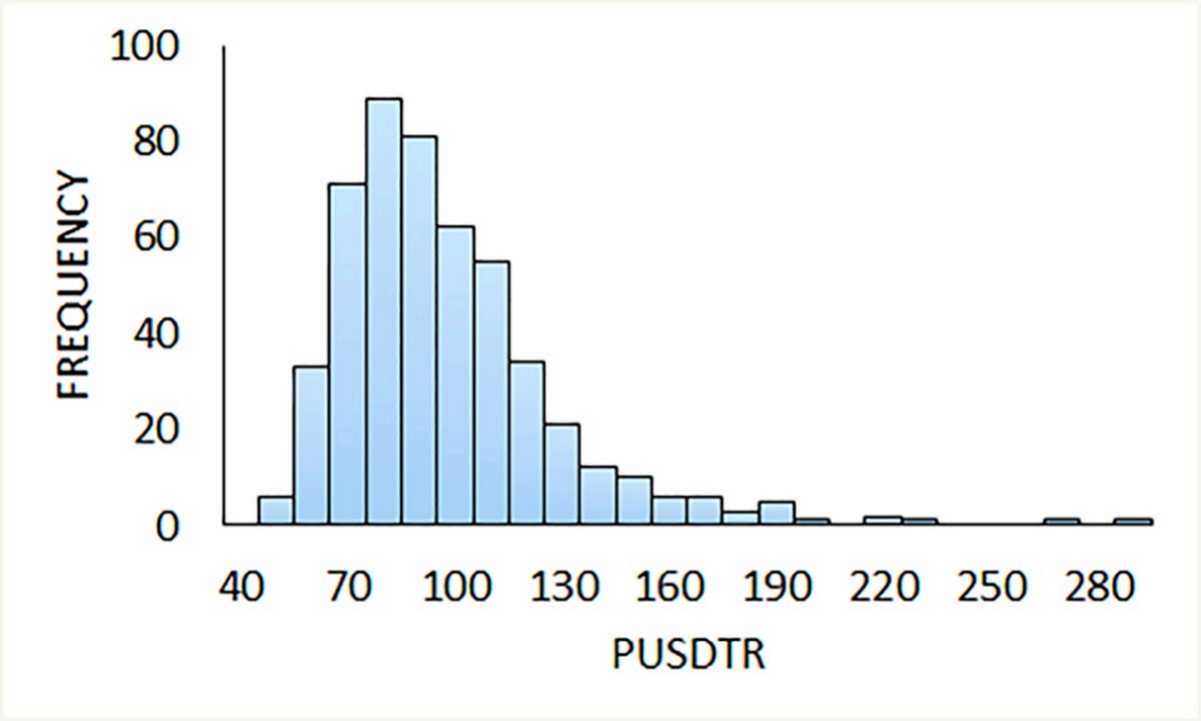
The peri-urban ↔ sylvatic exchange simulation results. This simulation calculates 500 different scenarios for a population of 100 000 humans that live in peri-urban areas and are in contact with the forest (with a density of 1 mosquito per human). The peri-urban ↔ sylvatic probability of dengue transmission ratio (PUSDTR) are measured. PUSDTR = (probability of dengue to be carried from the urban area to the forest)/(probability of dengue to be carried from the forest to the urban area).

We first used the mathematical model to examine the flow of DENV to and from the sylvatic environment. The simulation predicts a mean peri-urban/sylvatic transmission probability ratio of 92.5 with a standard deviation of 31.1. This means that the probability for a person to introduce an urban or peri-urban DENV sequence to the forest is 92.5 times greater than the opposite situation. Given that DENV prevalence in the mosquito is greater in the peri-urban areas than in the sylvatic ambit, it is more probable that a human vector introduces an urban or peri-urban DENV sequence to the forest than the contrary [58]. Also, the forest to urban transmission distribution follows a gamma distribution, like a Poisson process, which is typical of rare (unprovable) events (see Fig 2). These data suggest that although the sylvatic environment is often considered the source or reservoir of novel DENV serotypes, in fact peri-urban areas are significant sites of viral evolution and viral reservoirs which are more likely to disseminate DENV to the surrounding areas than vice versa, a position supported by experimental evidence [58].

### Novel serotype transmission is highly likely in the peri-urban setting

Since we found that rates of infection were higher in the peri-urban ambit and that viral transmission was more likely in the urban to sylvatic direction we decided to simulate the emergence of a new DENV serotype merely considering the urban transmission cycle of the virus. For this, we calculated the number of novel serotype transmission events with probability different from zero in the basal scenario and other scenarios with increased probability of mutation.

### Probability of consolidation constrains the rate of novel serotype emergence in dengue fever virus

**Fig 3:**
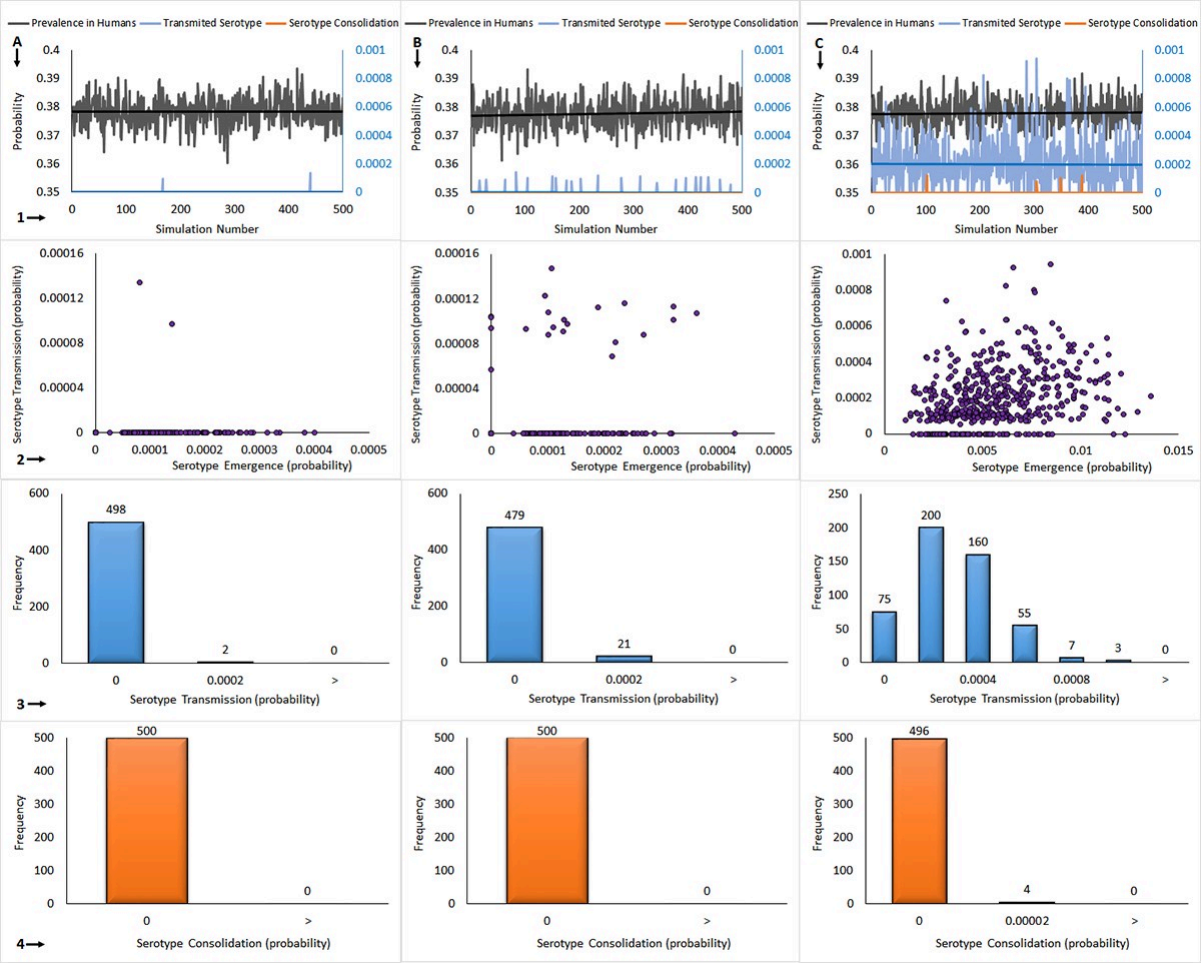
Modeling of novel DENV serotype emergence with a varying mutation rate and no vertical transmission. Column A: natural mutation rate, B: 100 x natural mutation rate, C: 1 000 x natural mutation rate. The graphs presented vertically are 1: 500 x (100 000 persons and 100 000 mosquitoes) simulation outcomes, 2: new serotype consolidation vs. new serotype transmission, 3: novel serotype transmission distribution, 4: novel serotype consolidation distribution.

In order to better understand the probabilistic factors controlling novel serotype emergence we next modeled the effect of varying mutation rate in the absence of vertical (mosquito-mosquito) transmission. We also identified consolidation events by considering the probability of a novel serotype emerging and then being successfully transmitted through one complete infection cycle, that is, from mosquito to human, and back to mosquito again. The results of this analysis are presented in Fig 3. The results shown in column A were calculated using the natural mutation rate value, while those in column B were the result of increasing the natural mutation rate by 100 times, and in C by 1 000 times. The scatter plots shown in the first row of graphics summarize the results of each of the 500 simulations, while the solid lines correspond to the mean value of these variables over the entire 500 simulations. We observed that the average value of the prevalence of DENV infection, indicated in black, (seroprevalence in virological terminology) calculated by our model are close to 40%, in line with the data reported in observational field studies [58]. The probabilities of new serotype transmission events are shown in blue while the probabilities of serotype consolidation events are shown in orange. The probabilities of transmission and consolidation events are calculated by dividing the number of transmission events divided by the 100 000 mosquitoes simulated in each cycle, giving the probability of novel serotype transmission or consolidation per mosquito per year. This value corresponds to the yearly probability of sufficient mutations accumulated in the E protein sequence so that a new virus serotype appears and infects a human, following a bite by an infected mosquito. Overall, no consolidation events were observed, except when the simulation was run at 1 000 times the natural viral mutation rate (Fig 3, Panel 1C). We observed that when using 1, 100, and 1 000 times the natural mutation rate value in the equations, the probabilities of new serotype transmission to humans were 1.77x10^-7^ [95% CI, 1.61x10^-7^–1.93x10^-7^] per year/per mosquito, 1.98x10^-7^ [95% CI, 1.13x10^-7^–2.94x10^-7^] per 100 years/per mosquito and 4.81x10^-7^ [95% CI, 1.88x10^-7^–1.07x10^-6^] per 1 000 years/per mosquito, respectively (average of 500 simulations). For each scenario the resulting serotype consolidation events had a probability of occurrence of 0, 0 and 2.15x10^-7^ [95% CI, 1.65x10^-7^–2.47x10^-7^] per 1 000 years/per mosquito; presenting only 6 observations of non-null probability when the mutation rate was multiplied one thousand times. This implies that a 1 000 year time-lapse is required to obtain probabilities of new serotype consolidation different from zero.

The second row of graphics in Fig 3 show the relationship between the probability of novel serotype transmission and novel serotype consolidation. We observed poor correlation between these two events, obtained in the 1 000 times normal mutation rate scenario (Fig 3, Panel 2C). In order to further clarify the role of mutation rate on novel serotype transmission and consolidation we created probability histograms of the number of simulations run that resulted in either novel serotype transmission (Fig 3, Row 3), or novel serotype consolidation (Fig 3, Row 4). At the normal mutation rate, 2 simulations resulted in transmission events, while increasing the mutation rate to 100 times or 1 000 times normal resulted in 21 and 425 transmission events, respectively (Fig 3, Row 3). In Fig 3, Row 4 we illustrate the occurrence of novel serotype consolidation. Only when the mutation rate was increased to 1 000 times normal was there evidence of consolidation of a novel serotype, with four simulations showing this result. These data suggest that novel serotype transmission is a common occurrence during the dengue infectious cycle, however, it is the process of consolidation that represents the largest probabilistic barrier to emergence of novel serotypes.

### Vertical transmission does not affect probability of novel serotype consolidation

**Fig 4:**
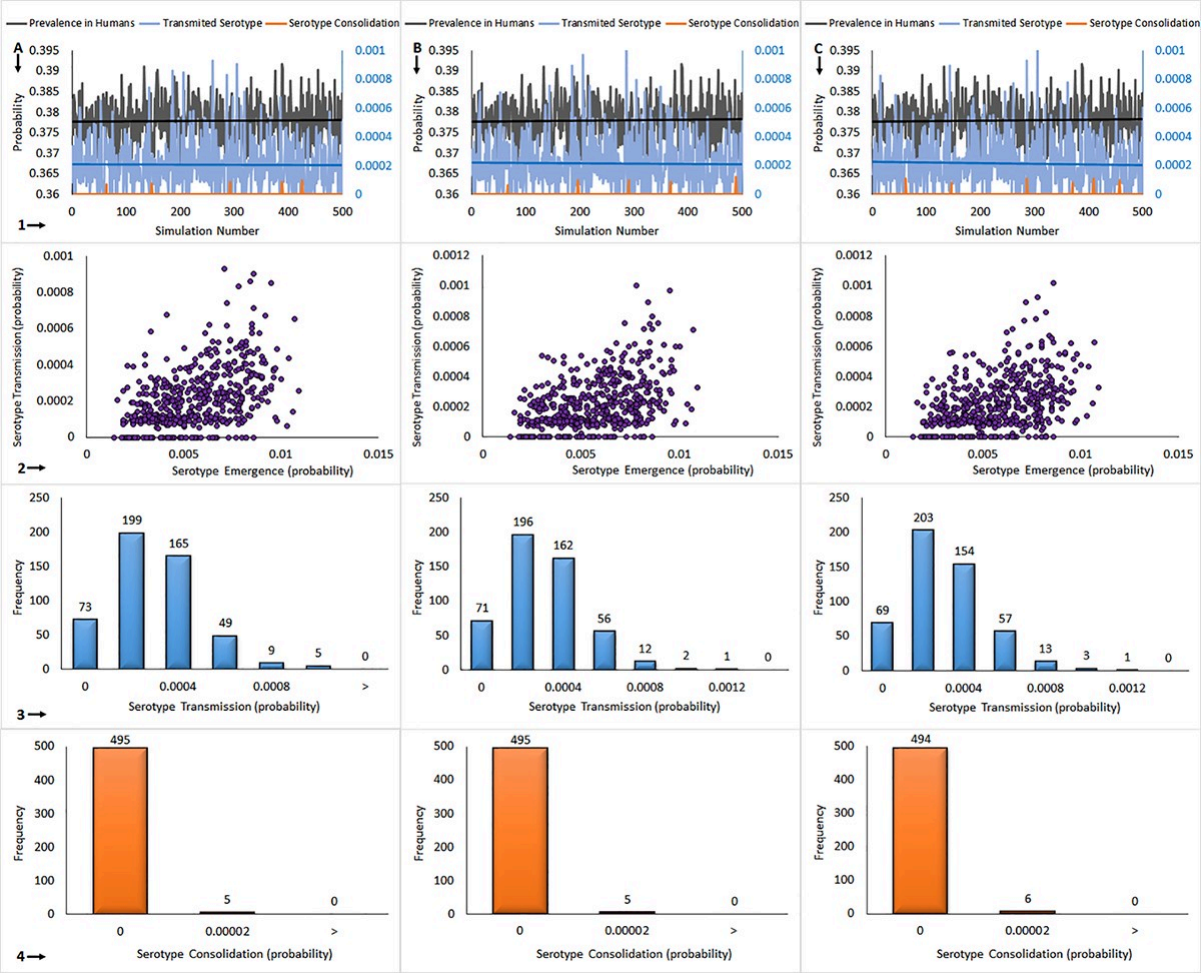
Modeling of the emergence of dengue virus presenting a new serotype with virus mutation rate 1 000 times normal, and with vertical transmission. Column A: 10% of vertical transmission, B: 30% of vertical transmission, and C: 50% of vertical transmission. The graphs represent vertically 1: 500 x (100 000 persons and 100 000 mosquitoes) simulation outcomes, 2: serotype emergence vs. serotype transmission, 3: serotype transmission distribution, 4: serotype consolidation distribution.

Vertical transmission of DENV from mosquito to mosquito has been documented via the deposition of infected eggs, a process known as transovarial transmission. This mechanism of transmission provides the virus with a longer time frame in which to mutate free of constraint by the immune response of the mammalian host. Furthermore, vertical transmission increases the number of mosquitos carrying a given viral serotype since they can be infected transovarially instead of acquiring infection through the probabilistically rare event of biting a human infected with a novel serotype. Therefore, we would initially hypothesize that vertical transmission would increase the likelihood of novel serotype transmission and ultimately novel serotype consolidation. In order to test this hypothesis, we ran our simulation under the same conditions as those illustrated in Fig 3 Column C, with 1 000 times the normal mutation rate, and with various rates of vertical transmission. We then set the fraction of mosquitos infected by vertical transmission at 10% (Fig 4, Column A), 30% (Fig 4, Column B), or 50% (Fig 4, Column C). In Row 1 of Fig 4 the results of these simulations are shown graphically, with the number of human infections shown in black, the number of novel serotype transmission events shown in blue, and the number of novel serotype consolidation events shown in orange. As the rate of vertical transmission was increased from 10% to 50% there was a slight, though not statistically significant, increase in the overall rate of human dengue infection (Fig 4, Row 1). Analysis of novel serotype transmission versus novel serotype consolidation probabilities showed the same poor correlation found in the model without vertical transmission (Fig 4, Row 2). Probability histograms illustrating the number of novel serotype transmission events under each of the vertical transmission scenarios are shown in Fig 4, Row 3. Increasing the rate of vertical transmission did result in a very slight increase in the number of novel serotype transmission events as expected, but the increase was so slight as to be essentially inconsequential (compare Fig 4, Panel 3A and Panel 3C; and Fig 3, Panel 3A versus Fig 4, Panel 3A). This was a rather surprising finding, since we expected that high rates of vertical transmission would have a profound effect on novel serotype transmission. We can infer from our results that DENV vertical transmission is only relevant for short time interval. Probability histograms demonstrating the effect of varying vertical transmission rates on incidence of novel serotype consolidation are shown in Fig 4, Row 4. The data show that there was no discernable effect of vertical transmission rate on novel serotype consolidation. Overall, the data indicate that, contrary to our initial hypothesis, vertical transmission has little effect either on the probability of novel serotype transmission or on novel serotype consolidation.

## Discussion

Variation in immunogenic epitope constitutes the hallmark of RNA virus evasion from the human adaptive immune system. Curiously, DENV presents few serotypes, even though numerous genotypes have been reported. Here, we used a Timed Markov Transmission Model to explore the effect of various factors on novel serotype emergence in dengue Fever Virus. Key factors explored in this work were the peri-urban ↔ sylvatic transmission ratio, viral mutation rate, the probability of initial novel serotype transmission versus novel serotype consolidation, and the influence of vertical transmission in the mosquito vector. An increased viral mutation rate, alternatively interpreted as longer timescales, did result in higher probabilities of novel serotype transmission and consolidation as expected. However, our model also revealed some rather unexpected predictions, suggesting the peri-urban area as the major site of viral evolution, little effect of vertical transmission on novel serotype emergence, and that while novel DENV serotypes evolve quite readily, consolidation represents the major probabilistic hurdle to their widespread emergence.

Commonly the sylvatic environment is considered a reservoir of DENV and the source of novel dengue virus serotypes in humans. This has been supported by data showing that the genomic sequences of sylvatic serotypes lay ancestral to the corresponding human DENV serotypes [11]. While this certainly indicates that the sylvatic environment is a reservoir for DENV serotypes it does not necessarily imply that those serotypes first evolved in the sylvatic context. In fact, the data presented here (Fig 2) suggests that novel serotype evolution and emergence are more likely to occur in the peri-urban setting and that resulting novel serotypes then pass to the sylvatic realm where they may be subsequently maintained. This has implications for viral control since it challenges the view of wild spaces as sites of novel viral emergence and directs us to also consider the peri-urban ambit as a rich location for viral evolution.

Our model provides valuable insight into the potential causes of the low rate of novel serotype emergence in DENV. The model provided an average probability of novel serotype transmission of 2.15x10^-7^ per mosquito per 1 000 years (Fig 3). These data indicate that in a pool of approximately 21 500 million mosquitos, a novel serotype transmission event could occur on a timescale of about one year. Given approximately 390 million human dengue virus infections worldwide each year, the observed low rate of novel serotype emergence becomes even more intriguing [59]. These observations are especially interesting since, as discussed above, there should exist strong positive selection for novel serotypes, yet very few are known. Critically, these data suggest that while novel serotype evolution and transmission is commonplace, the process of consolidation whereby chains of mosquito-human-mosquito infection occur is probabilistically very infrequent and forms the major constraint on novel serotype emergence.

While our model generally indicates a 1000-year timeframe is required for a non zero probability of novel serotype emergence, application of the above data to known rates of human dengue virus infection predicts novel DENV serotype emergence, on average, every 55.1 years (assuming 390 million of Dengue cases per year). This discrepancy is due to a basic assumption of the model that a single round of mosquito > human > mosquito transmission constitutes consolidation. For a novel serotype to emerge it must become sufficiently widespread that it is detected, so true “consolidation” requires many thousands of rounds of host alternation. Overall, the consideration of this data with respect to the approximately 390 million known yearly human DENV infections is a crude application of the derived probabilities at best.

On the whole, consideration of our findings in the context of the known number of human infections suggests the general validity of the model, while also exposing the need to uncover yet other factors affecting novel serotype consolidation. Additional considerations affecting novel serotype emergence include the effect of predation on, and reduced fecundity and fertility of infected mosquitos; as well as the effect of viral infectivity. One explanation for the still rather high novel serotype emergence rate obtained when applying the probabilities from the model to known human infection rates is the hypothesis that novel serotype emergence and consolidation are more common than experimentally observed. It is not unlikely that many novel serotypes consolidate briefly but lack the infectivity to be maintained over longer timescales. Given that very few persons affected by DENV have serology tests available to them, let alone being subject to viral isolation, serotyping, and genomic sequences it is not unexpected that many minor and transient consolidated serotypes could go undetected. The time of new serotype emergence obtained from this model is in line with the phylogenetic analysis of dengue serotype divergence [60].

Indeed, emergence of a fifth DENV serotype has been reported in India, with another novel serotype emergence reported in Malaysia, however, these serotypes failed to persist in the human or mosquito populations [61, 62]. In any case, it will be interesting to observe whether more novel and transient serotypes are detected as serotyping and sequencing abilities become more widely available, especially since these techniques have previously been, and continue to be, less accessible in DENV-affected countries and regions. The phenomenon of vertical (mosquito-mosquito) transmission had no obvious effect on the probability of novel serotype consolidation when included in our model (Fig 4). The model generally predicts novel serotype consolidation on a time horizon of 1 000 years, a result that is in line with phylogenetic studies of DENV. Large-scale virus sequencing of either dengue infected humans or vectors performed during an outbreak could potentially provide evidence that the consolidation step represent the major step in novel serotype emergence, giving us an insight of the sequences eliminated when passing from one host to the other.

The major finding of the current work is that consolidation is the major probabilistic roadblock to novel serotype emergence in DENV. Moreover, our findings suggest a rethinking of the role of the peri-urban and sylvatic environments as sites of viral evolution and novel viral emergence which are relevant not just to DENV but to many other extant and emerging viral diseases, such as West Nile Virus, Yellow Fever Virus, and Zika Virus [63]. Most importantly, the modeling presented here provides a useful basis for framing our assumptions regarding the transmission, evolution, and emergence of novel DENV variants, and can assist in management and control of this and other viral diseases of serious concern to public health.
